# Causes of morbidity and mortality in free-ranging Eurasian lynx (*Lynx lynx*) in Switzerland, 2000–2022

**DOI:** 10.1371/journal.pone.0344107

**Published:** 2026-03-24

**Authors:** Stéphanie Borel, Iris Marti, Francesco C. Origgi, Gary Delalay, Christine Breitenmoser, Samoa Zürcher-Giovannini, Caroline F. Frey, Walter Basso, Daniela Schweizer, Sonja Kittl, Marie-Pierre Ryser-Degiorgis, Saskia Keller

**Affiliations:** 1 Institute for Fish and Wildlife Health, Department of Infectious Diseases and Pathobiology, University of Bern, Vetsuisse Faculty, Bern, Switzerland; 2 Institute of Infectious Diseases, College of Veterinary Medicine, University of Messina, Messina, Italy; 3 Institute of Microbiology, Department of environment, constructions and design, University of Applied Sciences of Southern Switzerland, Mendrisio, Switzerland; 4 Foundation KORA Carnivore Ecology and Wildlife Management, Ittigen, Switzerland; 5 Institute of Parasitology, Department of Infectious Diseases and Pathobiology, University of Bern, Vetsuisse Faculty, Bern, Switzerland; 6 Division of Clinical Radiology, Department of Clinical Veterinary Medicine, University of Bern, Vetsuisse Faculty, Bern, Switzerland; 7 Institute of Veterinary Bacteriology, Department of Infectious Diseases and Pathobiology, University of Bern, Vetsuisse Faculty, Bern, Switzerland; University of Bucharest, ROMANIA

## Abstract

Health monitoring based on post-mortem examination is essential for the management of endangered animal species. This is especially true for reintroduced species living in small populations with low genetic diversity, such as the Eurasian lynx (*Lynx lynx*) in Switzerland. Thanks to systematic post-mortem examinations, the Institute for Fish and Wildlife Health (FIWI), University of Bern, has acquired a comprehensive view of the lynx health in Switzerland. This study provides an updated overview of the causes of morbidity and mortality in the Eurasian lynx in Switzerland from 2000 to 2022. A total of 346 necropsied lynx (found dead, euthanized, or culled) were included in this study, and a cause of death (COD) was identified in 318 of them (91.9%). Overall, the main COD was blunt trauma (n = 183, 52.9% - largely vehicular collision). Starvation, resulting from the separation of dependent juveniles from their mother, was the second most frequent COD (n = 63, 18.2%). Fatal infectious diseases were relatively low (n = 32, 9.2%). However, we documented some significant pathogens such as canine distemper virus (CDV) and metastrongyloid nematodes. Illegal killing was confirmed in 23 cases (6.6%). Of note, illegal killing is likely underestimated in this study, given that radio-collared lynx were found to be proportionally more often illegally killed than the unmonitored ones found by chance. Furthermore, most individuals were found to be affected at least by one non-specific, mild to moderate inflammatory process of unknown origin, such as interstitial pneumonia (n = 59) or interstitial nephritis (n = 25). Additionally, cardiac changes of variable severity were observed in 125 lynx, and severe soft tissue mineralization was detected in 10 individuals. The frequency of these findings warrants further investigation. Thus, this study confirms the importance of systemic post-mortem examination and general health surveillance of free-ranging Eurasian lynx in Switzerland, in support of translocation projects, conservation of the species, and to provide a better understanding of their pathologies.

## Introduction

In Central and Western Europe, Eurasian lynx (*Lynx lynx*) occur in small, reintroduced populations. These populations are still isolated and show poor genetic diversity, urging the need for pan-European conservation strategies. After its extinction in Switzerland at the end of the 19th century, the Eurasian lynx became protected by law in 1962 and was reintroduced in the 1970s [1]. Currently, three closely related yet genetically distinct populations exist in Switzerland, which are largely spatially isolated: (1) the Swiss Jura population (JUS) located within the Jura Mountains and extending into France, (2) the Alpine population (ALP), found in the central Alpine region of Southwestern Switzerland, and (3) the Northeastern population of Switzerland (NES), which was established between 2001 and 2008 with animals relocated from the other two populations [2].

Currently, there are an estimated 343 independent lynx in Switzerland [3]. Thus, lynx remain scarce and are classified as Endangered by the regional Red List Assessment [4].

Identifying the primary cause of death (COD) is essential for understanding population ecology, but is also important considering management and conservation perspectives [5]. The knowledge of cause-specific mortality is particularly relevant for endangered species, considering one of the most important actions to reverse a species’ decline is to mitigate the most significant causes of mortality through targeted efforts [6,7]. In Switzerland, health surveillance of the lynx populations consists of a combination of post-mortem examination of lynx found dead, culled, or euthanized [8], clinical examinations of lynx captured alive, and population monitoring (radio-telemetry and camera trapping) [9]. Since the early 2000s, post-mortem examinations of lynx in Switzerland have been carried out according to a standard necropsy protocol at the Institute for Fish and Wildlife Health (FIWI), Vetsuisse Faculty, University of Bern. This centralized system ensures a consistent assessment of morbidity and mortality causes and provides a valuable archive of biological material, including sample collection for further analyses (e.g., parasitology, virology, histopathology and genetics) and biobanking [10]. Since 2004, all lynx found dead, legally culled, or euthanized have been systematically submitted to the FIWI for post-mortem examination according to federal guidelines, forming the basis for comprehensive analyses of lynx mortality over time. The analysis of the COD of radio-collared animals is especially important as it allows examination of individuals whose carcasses would not necessarily have been found by chance without wearing radio-collars.

A first study on COD in free-ranging Eurasian lynx in Switzerland was carried out by Schmidt-Posthaus et al. [11], comprising 72 lynx submitted to the FIWI from 1987 to 1999. As in many other countries in Europe [12–14], the main documented COD were related to human activities, e.g., vehicular collisions and illegal killing.

The aims of the present study were to provide an update of the COD and diseases in Eurasian lynx in Switzerland from 2000 to 2022, according to different parameters such as sex, age category, and population. We wanted to assess whether these parameters could influence the pathologies and mortality rates of lynx. Specifically we tested for the following hypothesis: (A) we hypothesized that subadults would exhibit higher mortality rates due to vehicular collisions associated with dispersal movements; (B) that young lynx would be more susceptible to infectious diseases because of age-related vulnerabilities; (C) that lynx equipped with radio-collars would be reported more frequently as victims of illegal killing because their carcasses are more easily detected; and (D) that adult lynx would have higher rates of age-related degenerative cardiac changes.

## Materials and methods

### Study material

This retrospective study was performed with data from the FIWI necropsy database [8] and included a total of 346 animals, found by chance (n = 318) or collared lynx recovered after receiving a mortality signal (n = 28) that were submitted from 2000 to 2022. Submitted lynx were either found dead (n = 263), euthanized by a veterinarian (n = 17) or legally culled by game wardens (n = 66).

Lynx were mostly free-ranging, with few (n = 13) being caught in the wild and brought into wildlife care centers (WCC) or zoos (in Switzerland or abroad) where they later deceased. Most of them (n = 9) died or were euthanized between one and seven days after their admission into captivity from pre-existing problems. The remaining four had been placed in zoos and were submitted after their death [15].

Animals were categorized as juvenile, subadult or adult based on dentition, weight and body length [16,17]. Coordinates of the discovery location of the lynx (alive or dead) were used to determine the lynx population. For individuals that died in captivity (WCC or zoos), as well as for one lynx found dead in France (included in this study because its capture and collaring took place in Switzerland and its carcass was brought for post-mortem examination to the FIWI), the place of the initial capture has been considered.

### Post-mortem examination

Post-mortem examination for the determination of the COD included radiography, macroscopic examination, and histopathology. Morphometrical measurements of the body and inner organs as well as parasitological examination were performed for additional information on health status but are not part of the aim of this study and will not be discussed further, accordingly. Further ancillary testing (bacteriological, virological, or toxicological analyses) was carried out if deemed appropriate, in cases of suspicious macroscopic or microscopic changes.

In detail, examinations started with a latero-lateral full body radiography (n = 289/346, 83.53%) at the Division of Clinical Radiology, Department of Clinical Veterinary Medicine, Vetsuisse Faculty, University of Bern. This was followed by a standardized postmortem examination adapted to preserve the skeleton for taxidermic preparation. Stomach content was analyzed macroscopically for presence of anthropogenic food or foreign material. Samples of heart, lungs, liver, kidneys, spleen, genital organs, lymph nodes, thyroid, urinary bladder, stomach, small and large intestines, as well as from any tissue with macroscopic lesions, were collected for histopathology (n = 313/346, 90.86%). These tissues were fixed in 4% buffered formalin, embedded in paraffin, cut to 5 µm slices, stained with hematoxylin and eosin (HE) and observed by light microscopy. A modified Van Gieson’s stain was used to detect fibrous and vascular changes in the heart; other special stains were used if indicated. The brain was not examined because the skull, as well as the other bones, was left intact to preserve the skeleton for taxidermic preparation, except few exceptions. Deviation from the existing protocols and thus missing data were noted over the 23 years because of incomplete examinations.

### Laboratory analyses

Ectoparasites were mainly detected by direct visualization or by light microscopy following skin scraping in cases where mange was suspected. Ear mange and sarcoptic mange were consistently assessed for, but other ectoparasites were not systematically documented.

Endoparasites were mainly screened by coproscopy including sedimentation/zinc-chloride flotation (n = 299) and the Baermann funnel (n = 296) methods, performed at the Institute of Parasitology, Vetsuisse Faculty, University of Bern (IPB) [18]. Some endoparasites were detected incidentally via direct visualization or histology (n = 16), not through the methods outlined earlier.

Bacteriological examinations (n = 48) were initiated when evidence of a bacterial infection (e.g., splenomegaly, hepatomegaly, abscesses, wound infection etc.) were observed during post mortem examination, and carcasses condition was sufficient. Standard cultivation and identification were carried out using biochemical strips (in the early years) (API 20 E/NE), VITEK® 2 COMPACT (bioMérieux) or MALDI-TOF MS (Bruker) [19]. All analysis were conducted at the Institute of Veterinary Bacteriology, Vetsuisse Faculty, University of Bern (IVB).

Molecular analyses were performed in 19 lynx when characteristic clinical signs or macroscopic and/or microscopic lesions suggested a viral etiology. Conventional polymerase chain reaction (PCR) testing for canine distemper virus (CDV) (n = 14) was performed in-house at the FIWI in cases showing an interstitial pneumonia, or other lesion indicative of CDV-Infection. In one lynx presenting with necrosuppurative bronchopneumonia, additional PCR analyses were conducted for feline herpesvirus [20], calicivirus [21] and parvovirus [22] (each n = 1) at the Clinical Laboratory, Vetsuisse Faculty, University of Zurich, as these viruses are known to be associated with severe respiratory disease. Testing for feline immunodeficiency virus (FIV) (n = 4) was performed in live captive lynx shortly before euthanasia, in the framework of an international translocation program, already published [23].

Eight samples for toxicological screenings by gas chromatography-mass spectrometry (GC-MS) were sent to the Institute of Pharmacology, Toxicology and Pharmacy of the Ludwig-Maximilian University of Munich, Germany, and one case was examined at the Toxlab diagnostic laboratory in Lyon, France. All individuals selected for testing either exhibited abnormal behavior prior to their death or were found dead in proximity to other deceased animals, suggestive of a potential poisoning.

### Data analyses

All available data on the animals included in this study were compiled in an Excel table (Microsoft Excel 2010, Microsoft Corporation, Redmond, Washington, USA), including date and season of discovery or capture, age, weight, sex, geographical origin, population (ALP, JUS or NES), circumstances of findings and body condition. Body condition was scored as poor, moderate or good based on the amount and appearance of muscular mass and subcutaneous and visceral adipose tissue. Finally, post-mortem findings were reported and divided into main and incidental findings. Main diagnosis represents the primary COD or reasons for culling/euthanasia and were categorized as 1) non-infectious diseases, including traumas (vehicular collision, other anthropogenic traumas, and miscellaneous traumas), mother’s separation (i.e., juveniles dependent from the mother, in moderate or poor body condition, with no other findings of significance observed), illegal killing, legal killing (after repeated livestock predation) and miscellaneous diseases, or 2) infectious diseases (parasitic, bacterial, or viral).

Relevant incidental findings, which represent concomitant morbidities, are later discussed and have been categorized by organ system. Focus on the incidental findings was arbitrarily oriented based on the frequency of the repeated occurrence and on the estimated clinical relevance of the lesion observed on the basis of its extent and severity. Classification into main or incidental findings, as well as the significance of the incidental findings. was based on the known or presumptive consequences of these changes on the animal’s health, as well as on the pathologist on duty and the first authors’ expertise.

### Statistical analyses and software

To assess factors influencing the probability of discovering dead lynx in Switzerland, Chi squared tests were computed with R 4.4.2 (R Core Team,2015. R: A language and environment for statistical computing, Vienna, Austria. URL: http://www.r-project.org/).

To assess factors influencing certain diseases, linear regressions were fitted using R, packages dplyr 1.1.4 [24] and parsnip 1.2.1 [25] and the function glm from the stats package. Information on the regression can be found in the supplementary material S1 File.

We tested the correlation of lynx population (ALP, JUS and NES), age category (juvenile, subadult, adult), sex (female, male) and wearing a radio-collar with selected CODs (vehicular collisions, illegal killing, miscellaneous traumas) and incidental findings (degenerative changes related to the heart (arteriosclerosis, myocardial fibrosis and/or muscle fiber degenerations), ear mange, lungworms), for which hypotheses had been put forward.

For each variable, the category with the highest number of occurrences was used as a baseline for comparisons, except for age where adult was used as baseline. Additionally, JUS was used as baseline for the correlation of the lynx population with the presence of ear mange, as it was significantly different when compared to ALP and NES. The level of significance was set at p < .05. For multiple linear regressions, the q value was also reported and its significance was also set at p < .05.

Linear regressions were also used for pairwise comparisons between COD of radio-collared lynx and dead lynx found by chance, except for viral diseases as COD. In this case, Fisher’s exact test was computed in R, as only 4 resp. 0 cases of viral diseases were found for lynx with respectively without radio-collar.

Chi square test was computed in R for comparisons between main COD listed in the study of Schmidt-Posthaus et al. [11] and in this study.

Figures were created in R using packages ggplot2 4.0.1 [26]. For the map, in addition to package ggplot2, packages raster 3.6–32 [27], sf 1.0–23 [28–29], ggspatial 1.1.10 [30] and terra 1.8–86 [31] were also used. The shapefiles for Swiss and cantonal boundaries and for lakes and rivers as well as the raster data for Swiss relief were all obtained from swisstopo.

Furthermore, any grammatical doubts throughout the manuscript were addressed using the DeepLWrite Grammar Checker (https://www.deepl.com/de/write, accessed on 1 June 2024).

## Results

### Animal demographics

An overview of the animals age, sex and population distribution is listed in Table 1.

**Table 1:** Determined age category, sex, and population for each free-ranging Eurasian lynx submitted to the Institute for Fish and Wildlife Health, Switzerland, 2000-2022.

	Juv (M/F/U)	SA (M/F/U)	Ad (M/F/U)	Total (M/F/U)
**ALP**	109 (54/50/5)	30 (18/10/2)	45 (20/18/7)	184 (92/78/14)
**JUS**	65 (28/35/2)	29 (19/9/1)	36 (16/17/3)	130 (63/61/6)
**NES**	20 (8/11/1)	6 (3/2/1)	6 (4/2/0)	32 (15/15/2)
**Total**	194 (90/96/8)	65 (40/21/4)	87 (40/37/10)	346 (170/154/22)

Juv, juvenile; SA, subadult; Ad, adult; M, male; F, female; U, unknown sex; ALP, Alpine population; JUS, Jura Mountain population; NES, Northeastern population of Switzerland.

**Fig 1:**
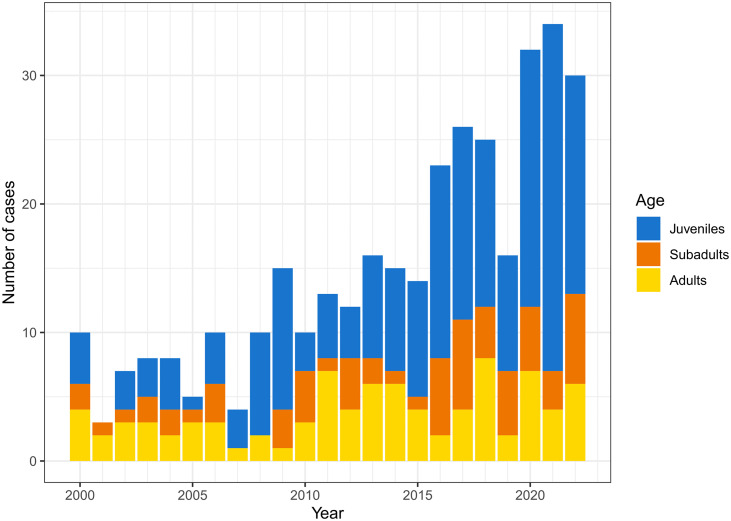
Number of lynx euthanized, legally culled or found dead per year in Switzerland, 2000-2022, according to age category (blue, juveniles; orange, subadults; yellow, adults).

The number of cases examined during these 23 years increased gradually each year and varied from 5 to 34 (in 2021) per year (Fig 1).

The submitted animals included adults (n = 87, 25.1%), subadults (n = 65, 18.8%) and juveniles (n = 194, 56.1%). There was no significant difference between the number of submissions of adults and subadults (*X*^*2*^ (1, N = 152) = 3.18, p = .074), but juvenile lynx were submitted significantly more often than subadults (*X*^*2*^ (1, N = 259) = 64.25, p < .001) and adults (*X*^*2*^ (1, N = 281) = 40.74, p < .001use).

**Fig 2:**
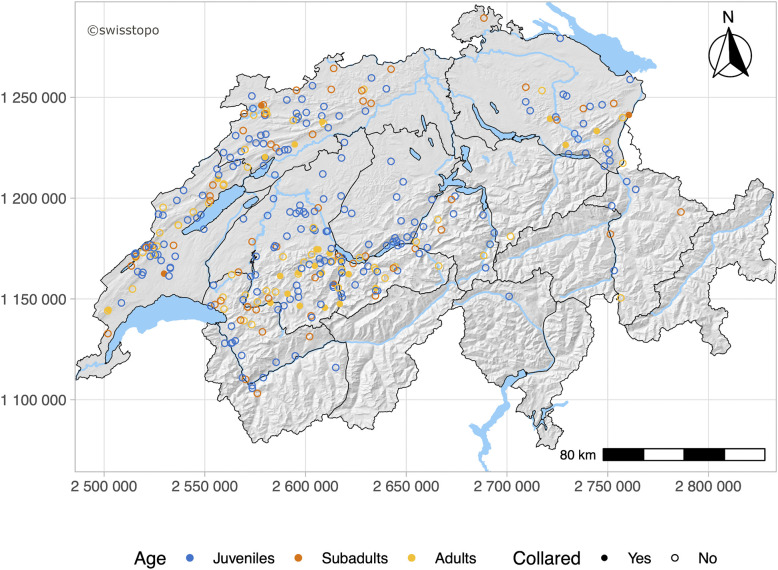
Map of Switzerland. Black lines indicate borders of current lynx management compartments. Geographical origin of all lynx involved in the study including animals which were euthanized, culled, or found dead (blue, juveniles; orange, subadults; yellow, adults).

The geographical origin of the cases submitted represented overall the three populations JUS, ALP and NES (Fig 2). Lynx originated significantly more often from ALP (n = 182, 52.6%) than from the JUS (n = 133, 38.4%, *X*^*2*^ (1, N = 315) = 7.62, p = .006) and the NES (n = 31, 9.0%, *X*^*2*^ (1, N = 213) = 107.05, p < .001).

A two-sided binomial test could not find a significant difference in the sex-ratio, with similar numbers for males (n = 170) and females (n = 154) (*X*^*2*^ (1, N = 324) = 0.79, p = .374). The sex of 22 carcasses could not be determined due to traumatic tissue loss or advanced decomposition.

### Causes of death

A COD could be identified in 318 cases (91.9%). For the remaining animals (n = 28, 8.0%), the COD could not be determined mainly due to submission of carcasses in an advanced state of decomposition. Classification of the COD, organized by age category and sex, is summarized in Table 2.

**Table 2:** Overview of the main cause of death or reason for culling or euthanasia diagnosed in free-ranging Eurasian lynx submitted to the Institute for Fish and Wildlife Health, Switzerland, 2000-2022.

	Juv			SA			Ad			TOTAL
	M	F	U	M	F	U	M	F	U	
**Non-infectious**										**286 (82.7%)**
**Traumas**	**44**	**48**	**2**	**34**	**16**	**0**	**23**	**17**	**0**	**183 (52.9%)**
Vehicular collisions	36	33	1	33	12	0	15	13	0	143
Others anthropogenic trauma	0	3	0	0	2	0	0	1	0	6
Miscellaneous trauma^1^	8	11	1	1	2	0	8	3	0	34
**Mother’s separation**	**32**	**31**	**0**	**0**	**0**	**0**	**0**	**0**	**0**	**63 (18.2%)**
Starvation	30	24	0	0	0	0	0	0	0	54
Legally culled orphans in good body conditions^2^	2	7	0	0	0	0	0	0	0	9
**Illegal killing**	**1**	**5**	**2**	**1**	**1**	**2**	**3**	**8**	**2**	**23 (6.5%)**
Gunshot	1	3	0	1	1	2	2	6	1	17
Poisoning	0	1	0	0	0	0	1	2	0	4
Others^3^	0	0	1	0	0	0	0	0	1	2
**Legal killing (livestock predation)**	**0**	**0**	**0**	**0**	**0**	**0**	**4**	**2**	**0**	**6 (1.7%)**
**Miscellaneous disease** ^4^	**3**	**4**	**0**	**0**	**1**	**0**	**3**	**0**	**0**	**11 (3.2%)**
**Degenerative diseases**	**3**	**3**	**0**	**0**	**1**	**0**	**2**	**0**	**0**	**9**
Heart failure	0	0	0	0	0	0	2	0	0	2
Severe soft tissue mineralisations	3	3	0	0	1	0	0	0	0	7
**Neoplasia**	**0**	**0**	**0**	**0**	**0**	**0**	**1**	**0**	**0**	**1**
**Malformation**	**0**	**1**	**0**	**0**	**0**	**0**	**0**	**0**	**0**	**1**
**Infectious**										**32 (9.2%)**
**Bacterial** ^5^	**7**	**7**	**0**	**1**	**1**	**0**	**3**	**1**	**0**	**19 (5.5%)**
**Pneumonia**	**3**	**3**	**0**	**0**	**1**	**0**	**2**	**0**	**0**	**9**
*Strept. canis*	0	1	0	0	0	0	0	0	0	1
*Staph. aureus* and *Strept. canis*	1	0	0	0	0	0	0	0	0	1
*Strept. equi* subsp. zooepidermicus, *Staph. aureus* and *P. multocida* subsp. multocida	0	1	0	0	0	0	0	0	0	1
*Strept. pseudintermedius* and *Strept. canis*	0	0	0	0	0	0	1	0	0	1
*Staph. schleiferi* subsp. *coagulans* and *Strept. canis*	0	1	0	0	0	0	0	0	0	1
Bact. analysis not done or inconclusive^5^	2	0	0	0	1	0	1	0	0	4
**Infected wound** (septicemia)	**1**	**1**	**0**	**0**	**1**	**0**	**1**	**0**	**0**	**4**
*Clostridium* sp.	0	0	0	0	0	0	1	0	0	1
*Clostridium haemolyticum*	1	0	0	0	0	0	0	0	0	1
*Staph. aureus, Staph. felis* and *Past. multocida* subsp. multocida	0	0	0	0	1	0	0	0	0	1
*Strept. canis* and *Ecoli*	0	1	0	0	0	0	0	0	0	1
**Systemic disease**	**3**	**2**	**0**	**1**	**0**	**0**	**0**	**0**	**0**	**6**
*Yersinia pseudotuberculosis*	2	1	0	0	0	0	0	0	0	3
*Listeria* sp.	0	0	0	1	0	0	0	0	0	1
Sterile analysis (myocarditis)^5^	1	0	0	0	0	0	0	0	0	1
Bact. analysis not done (septicemia)^5^	0	1	0	0	0	0	0	0	0	1
**Parasitic** (mange)^6^	**0**	**0**	**0**	**1**	**1**	**0**	**2**	**5**	**0**	**9 (2.6%)**
**Viral**	**0**	**0**	**0**	**1**	**0**	**0**	**2**	**1**	**0**	**4 (1.2%)**
*Canine distemper virus* (CDV)	0	0	0	0	0	0	1	0	0	1
*Feline immunodeficiency virus* (FIV)	0	0	0	1	0	0	1	1	0	3
**Undeterminable** ^7^	**3**	**2**	**6**	**2**	**1**	**2**	**0**	**5**	**7**	**28 (8.1%)**
**Total**	**90**	**96**	**8**	**40**	**21**	**4**	**40**	**37**	**10**	**346**

Juv, juvenile; SA, subadult; Ad, adult; M, male; F, female; U, unknown sex.

^1^This category includes lynx that died from natural trauma (n = 12) or trauma whose exact origin could not be demonstrated (n = 22).

^2^Nine Juveniles, still in good body condition, were found without their mother near human settlements and culled therefore.

^3^Others refers to an individual whose four paws were sent anonymously to a cantonal authority and to a juvenile mistaken for red fox and killed by blunt trauma to the head.

^4^This category includes lynx that died of degenerative diseases (n = 9), neoplasia (n = 1) and presumptive malformation (n = 1).

^5^Nineteen cases of fatal bacterial infections were reported, but only 13 were confirmed by culture, either because bacteriological cultures were not performed (n = 2), analyses were negative (sterile tissues, n = 3) or inconclusive due to the advanced decomposition of the carcass (n = 1). In these cases, diagnosis is based on histopathology.

^6^Death by cachexia or legal culling.

^7^The COD could not be determined due to carcasses in advanced state of decomposition (n = 23). The reason for the apparent poor health of the three remaining lynx remains unknown (two of them were found with severe neurological disorders and culled, and a third with presumptive locomotor difficulties).

### Non-infectious diseases

#### Vehicular collision.

The most frequently diagnosed COD was blunt trauma due to vehicular collisions (n = 143/346, 41.3%), caused by road traffic and trains. All animals showed hemorrhages in skin, subcutaneous tissues, musculature, thoracic and/or abdominal cavities. Multiple fractures of the limbs (n = 78), skull including jaws (n = 56), pelvis (n = 40), spine (n = 39) and/or ribs (n = 31) were commonly associated with trauma. Lacerations of the affected musculature and internal organs were frequently observed (n = 83). Traumatic exophthalmos, bone dislocations and tissue loss were also sporadically reported.

Subadult lynx were significantly more often affected by vehicular collisions than adults (odd ratio = 4.04; 95% CI [1.81, 9.34]; p < .001; q = .003). Males were more often affected than females (odd ratio = 1.71; 95% CI [1.04, 2.83]; p = .035; q = .047). JUS lynx were affected more often than ALP lynx (odd ratio = 2.96; 95% CI [1.78, 4.99]; p < .001; q < .001) and NES lynx less than ALP lynx (odd ratio = 0.32; 95% CI [0.10, 0.88]; p = .040; q = .047). The proportion of deaths caused by vehicular collisions has increased significantly since the first study from Schmidt-Posthaus et al. [11] which reported 34% between 1987 and 1999 (*X*^*2*^ (1, N = 418) = 9.79, p = .002).

#### Other anthropogenic traumas.

Six individuals died of causes associated to other human activities. One juvenile was found dead trapped in an electric fence surrounding a flock of sheep; another juvenile had to be euthanized following severe dental injuries that were self-inflicted in captivity during a rehabilitation attempt; a third was killed by a conspecific after being placed in a zoo and a last one died within its rehabilitation enclosure where it was found trapped behind a wooden fence. Additionally, two adult lynx died after immobilization for research and management purposes. The first one was found drowned the day after recovery from anesthesia, and the second one broke a defective trap and escaped with a leghold snare around its left front limb. This lynx was later found in a chicken coop with severe leg injuries encompassing necrotic skin, tendon and musculature and had to be culled.

#### Miscellaneous traumas.

A traumatic COD not directly linked to human activities was recorded for 34 individuals. Miscellaneous traumas, including traumas of natural origin (n = 12) and traumas of unknown origin (n = 22), represent the third common COD.

Six of these cases were probably related to a fall, given that the animals were found at the bottom of cliffs. These animals showed hemorrhages in skin, subcutaneous tissues, musculature, thoracic and/or abdominal cavities associated with multiple bone fractures. Two lynx were suspected to have died buried in an avalanche; one lynx was found trapped between two branches in a tree; one lynx was found in a well where it had presumptively drowned, given the severe pulmonary emphysema with alveolar foreign material; one juvenile died of a severe perforating gastric ulcer following the ingestion of a hoof; and the last lynx appeared to have choked on a hull of beechnut just after having predated a chamois. The hull was found obstructing the distal portion of the larynx, with diffuse congestion of the mucosa of the glottis and trachea, as well as a moderate hemopericardium and a moderate to severe hemothorax. Histologically, this lynx presented moderate to severe arteriosclerosis with multifocal interstitial myocardial fibrosis.

In 22 animals, the traumatic origin could not be further identified. These animals were found injured or dead, suffering from fractures and/or severe hemorrhages without immediate proximity to a road. However, it cannot be excluded that these animals were injured in a traffic accident and then moved until death and discovery. Six of them were wearing radio-collars, which led to their discovery. One radio-collared adult was found dead in a river 13 days after capture. In the vicinity of its GPS locations, several baits containing nicotinamide, nicotine and caffeine were recovered, however, testing of the individual’s blood was negative. Post-mortem examination revealed a mild encephalitis, pulmonary edema and poor body condition of unknown clinical relevance and without conclusive etiology. The COD remained unknown.

#### Mother’s separation.

The separation of dependent lynx from their mothers was identified as the direct COD of 63 juveniles (n = 63/346, 18.2%) and is the second most frequent COD in this study.

Among these 63 juveniles, 54 individuals were found dead or culled due to poor body condition, while nine were found alive without their mother near human settlements and legally culled therefore. The latter were still in good body condition with no pathological changes of significance observed during postmortem examination.

Injured or sick juveniles in moderate to poor body condition did not qualify as orphans here, as it was not possible to determine with certainty whether the emaciation (and therefore the supposed separation from the mother) occurred before or after the trauma or illness.

#### Illegal killing.

**Fig 3:**
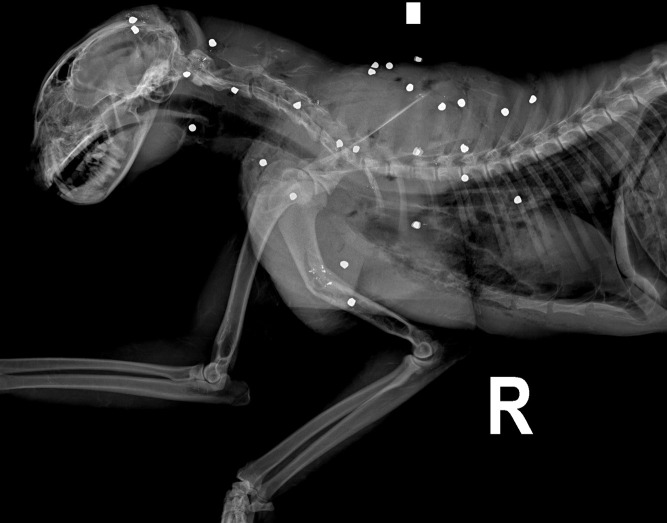
Right lateral radiograph of the cranial body of a juvenile lynx. Large amount of well-defined metal opaque, around 6 mm in diameter rounded fragments distributed along the thoracic wall, left forelimb, left side of the neck and caudal aspect of the head, associated with multifocal subcutaneous emphysema indicative of a gunshot wound and thus illegal killing. Comminuted diaphyseal fracture of the left humerus.

Illegal killing was the fifth most common COD (n = 23/346, 6.5%). Illegal killing was done either by deliberate shooting (n = 14) (Fig 3), poisoning (n = 4), because of presumptive species confusion (n = 4) or by unknown means (n = 1). The death by unknown means refers to an individual whose four paws were sent anonymously to one of the cantonal authorities and illegal activity is strongly suspected. In cases of species confusion, individuals were apparently mistaken for red fox and killed by blunt trauma to the head (n = 1), gunshot in the darkness of a stable (n = 1) or by gunshot in the forest during hunting (n = 2).

Poisoning by chloralose (n = 3) and cyanide (n = 1) was identified to be the COD for four lynx, in three different incidences. Chloralose was detected in two incidences: in the stomach content of two dead lynx (a radio-collared female and one of its cubs) and in the urine of a culled individual. In the latter case stomach and intestine contents, liver and kidney tested negative before urine was sent for examination. Also, despite the substance not being found in the stomach content of the female’s second cub, poisoning was strongly suspected (as the chloralose could not be conclusively proven, this individual was classified in the “undeterminable” cases). The culled individual which tested positive for chloralose in urine, was found by chance near a dead chamois, a fish, pieces of cork and latex gloves. It was apathic, trembling, and unresponsive, leading to its culling by a game warden. Four dead red foxes had been discovered in the same area weeks earlier. Cyanide was found in one incident, in the stomach contents of an uncollared female accompanied by its cub, found near a dead chamois, in a convulsive state, unable to stand up and having severe breathing difficulties. Both mother and cub were culled by the game warden. No substance was detected in the urine of the cub, but being found next to its poisoned mother and in absence of pathological lesions, an ingestion of cyanide was also strongly suspected (this cub was classified in the “undeterminable” cases).

In addition to the 14 lynx confirmed to have been deliberately killed by gunshot, 13 other lynx were found to have shotgun pellets or lead fragments embedded in their tissues. These non-lethal injuries were discovered incidentally during radiography or during necropsy examination, even though these lynx had died from other causes. In summary, illegal killing attempts amounted to 38 cases, including deliberate shooting, cases of non-fatal illegal shooting, unknown means and poisoning. Juvenile lynx and subadults were significantly less affected by illegal killing attempts than adults (odd ratio = 0.17; 95% CI [0.07, 0.41]; p < .001 resp. odd ratio = 0.34; 95% CI [0.11, 0.93]; p = .048).

#### Legal killing.

Lynx which predate more than 15 domestic animals within 12 months are allowed to be shot by cantonal authorities according to the Swiss hunting law [32]. During the 23 years, six lynx have been legally culled for this reason (6/346, 1.7%). Postmortem examination revealed no significant pathological lesions.

#### Miscellaneous diseases.

Few animals died of degenerative diseases (n = 9), neoplasia (n = 1) or presumptive malformation (n = 1). Seven animals, including six juveniles and one subadult, died with severe soft tissue mineralisations in kidney, stomach, and lung, indicative of metastatic mineralization. One of the juveniles had been admitted to a WCC and a blood test revealed signs of uremic syndrome (high urea and creatinine), as well as hyperphosphatemia indicative of renal failure. The toxicological analysis of stomach content of one of the individuals (toxicological screening and specific test for ethylene glycol presence), was negative. In three additional juveniles which died of other causes (vehicular collision, traumas of unknown origin and septicemia) identical soft tissue mineralisations in kidneys, stomach, and lungs were present. It is highly suspected that those individuals were highly debilitated and would have succumbed as the severity of the mineralization were incompatible with life. All affected animals were found in proximity to human settlements, directly in a village or within 250 m of a dwelling, between 2012 and 2022. Of the ten individuals affected, all but one came from the NES population.

Two radio-collared adult male lynx, 7 and 14 years old, respectively, died of heart failure. Both were in good nutritional and health status at the time of capture, despite a systolic heart murmur (grade 2) heard on clinical examination. The first lynx was immobilized four times within the framework of the translocations project to the NES population between September 1999 and May 2003 and was found dead next to a marmot a few weeks after the last capture. It presented with a severe generalized circulatory failure characterized by subcutaneous edema, hydrothorax, hydropericardium, ascites and peritonitis. Main histopathological findings were myocardial arteriosclerosis and fibrosis and centrilobular liver fibrosis with congestion. A subaortic stenosis was additionally diagnosed. The second lynx was culled in 2012 because of abnormal behavior and severe emaciation. Postmortem findings, clearly indicative of an chronic heart failure are described by Ryser et al. (2021), case 4 [33].

**Fig 4:**
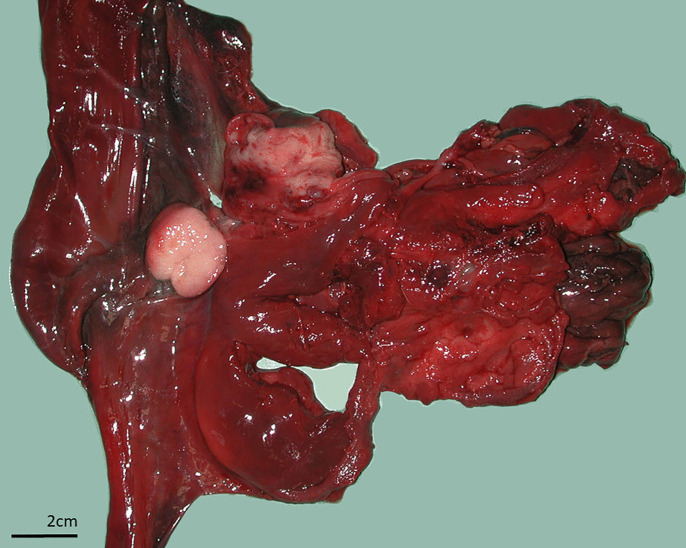
Proximal part of duodenum of lynx with lymphoma. Proliferation of the intestinal mucosa in the form of polyp, enlarged lymph node cut in two.

Only one animal died due to a neoplasia: a 12-year-old male died of a lymphoma affecting the mesentery and lymph nodes whose mass led to rupture of a large vessel and, consecutively, to hemothorax and hypovolemic shock (Fig 4).

One juvenile was taken into a WCC to be rehabilitated where it died secondary to a presumptive diaphragmatic defect. After a week, it presented acute clinical signs such as vomiting, increased respiratory rate and a comatose state. It was found dead the day after in the enclosure. The postmortem examination revealed a diaphragmatic defect and a diaphragmatic hernia of the stomach in the esophageal hiatus area, compatible with a congenital defect even though a trauma cannot be ruled out entirely.

#### Infectious diseases.

Infectious diseases were the fourth most frequent COD (n = 32/346, 9.2%) (Table 2), with nine cases of mange, 19 bacterial and four viral infections.

#### Parasitic diseases.

**Fig 5:**
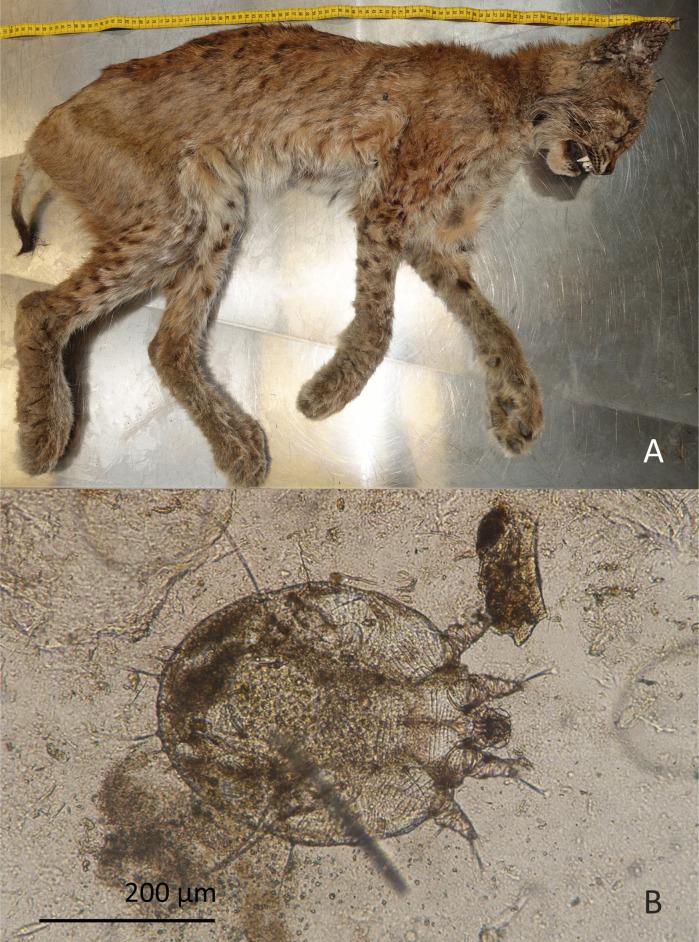
Lynx with severe sarcoptic mange. **(A)** Presence of crusts and hypotrichosis mainly in facial area, ears, legs, and tail. **(B)** Female *Sarcoptes scabiei* mite microscopically detected in skin lesions following skin scrapings.

Macroscopically visible skin lesions were attributed to sarcoptic mange in a total of 12 individuals from various origins (6 ALP, 4 JUS, 2 NES) and different age categories (1 juvenile, 3 subadults, 8 adults) submitted from 2002 to 2021 based on the identification of *Sarcoptes scabiei* mites in skin scrapings. For nine of them, mange was considered as primary COD; seven died of cachexia and two were culled because of severe macroscopic skin lesions compatible with mange (Fig 5). Most frequent non-lethal parasitic infections are listed within the incidental findings. No deaths were directly linked to endoparasites.

#### Bacterial diseases.

Nineteen cases of fatal bacterial infections were reported, of which 13 were confirmed by culture (Table 2). The other six cases remained presumptive either because confirmatory analyses were negative (sterile tissues, n = 3), inconclusive due to the advanced decomposition of the carcass (n = 1), or because bacteriological investigations were not performed (n = 2). In these cases, diagnosis was made by histopathology based on the presence of degenerated neutrophils associated to intralesional coccobacilli within lung (n = 4) and/or heart, muscle, spleen, liver and intestine (each n = 1).

Within bacterial fatalities, bacterial pneumonias were most observed (n = 9, including six juveniles) commonly caused by various Streptococci or Staphylococci with few co-infections (Table 2). One lynx that died of a suppurative pneumonia also had a severe chronic generalized myositis, but culture was negative.

In four cases, severely infected wounds were considered to be fatal by having developed into septicemias. A lynx exhibited several bites to the thorax and pelvis, likely caused by a co-specific (intraspecific aggression). A second one had a necrotizing osteomyelitis of the nasal bone, potentially caused by a skin injury. A third one had a chronic comminuted fracture of the humerus associated with dermatomyositis, osteomyelitis, and muscle fibrosis. The last individual had an old eye injury, a severe inflammation of the cornea (keratitis), extending along the optic nerve (periophtalmitis und perineuritis) leading to a meningoencephalitis. Details of bacteria involved are listed in Table 2.

**Fig 6:**
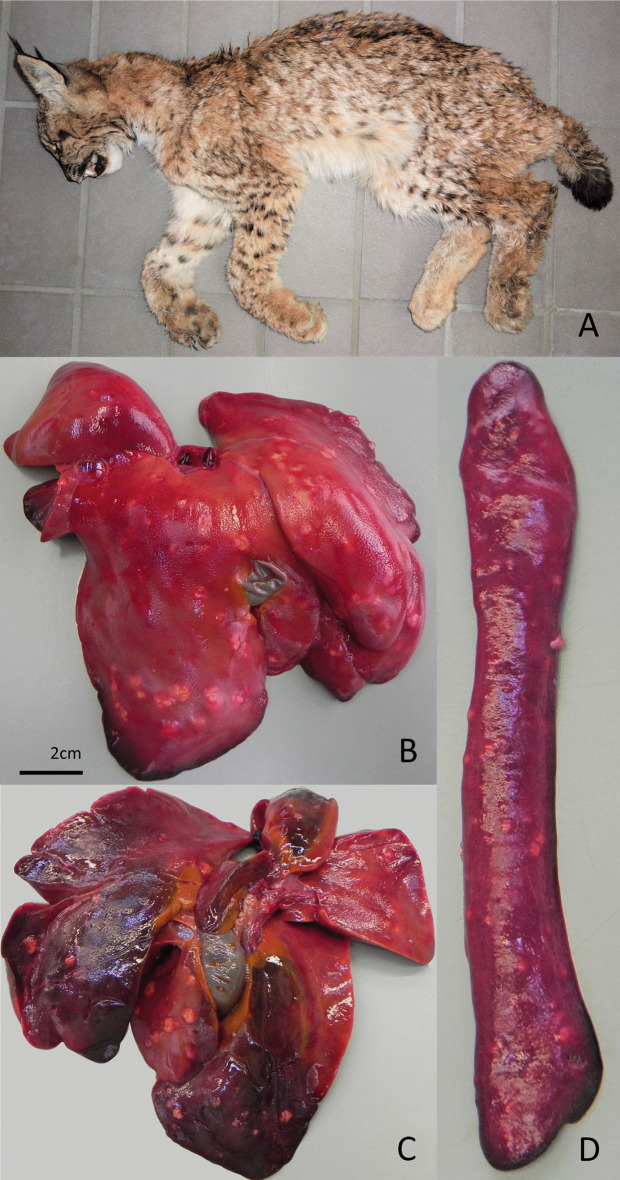
Juvenile lynx euthanized with pseudotuberculosis. **(A)**. Multiple nodular necrotic foci on the facies diaphragmatica of the liver **(B)**, on the facies visceralis **(C)** and on the spleen **(D)**.

*Yersinia pseudotuberculosis* was isolated from the liver and/or spleen of three animals causing their death (Fig 6).

One lynx died of listeriosis with *Listeria monocytogenes* isolated from the spleen by culture [34]. Another lynx died of a septicemia following intestinal invagination and rectal prolapse. This animal was found dead, and no bacteriological analysis was performed. One other lynx died of severe purulent myocarditis though culture was sterile.

#### Viral diseases.

Nineteen virological tests were carried out following suspicions of viral diseases, and 5 were positive. Three adults were euthanized following being tested positive for FIV during routine examinations within a translocation project [23]. Two other lynx were infected with canine distemper virus (PCR and IHC positive), in 2010 [35] and 2011 respectively. The last one was hit by a train and was still in good body condition. This viral infection was therefore considered to be the COD only in the first individual. However, both individuals showed interstitial pneumonia (mild and moderate, respectively) and mild necrotizing, lymphocytic to histiocytic myocarditis.

### Incidental findings

#### Unspecific inflammatory or degenerative changes.

During the 23 years of lynx post-mortem examinations, many nonspecific inflammatory or degenerative changes have been found with no direct association to the COD and were therefore considered as incidental findings. Most common observed incidental findings according to age categories and populations of origin are listed in Table 3. Further incidental findings affecting only a few individuals are not listed.

**Table 3:** Recurrent incidental findings recorded in lynx, according to age category and population of origin (in parenthesis: ALP/JUS/NES) submitted to the Institute for Fish and Wildlife Health, Switzerland, 2000-2022.

Main incidental findings	Juv	SA	Ad	TOTAL
**Cardiovascular system (heart)** ^1^				**125 lynx**
Degenerative heart conditions only	20 (11/9/0)	16 (5/10/1)	36 (18/13/5)	72 (34/32/6)
Cardiac inflammations only	17 (10/6/1)	8 (4/4/0)	7 (3/3/1)	32 (17/13/2)
Both (degenerative heart conditions and cardiac inflammations)	3 (1/2/0)	5 (3/1/1)	10 (5/5/0)	18 (9/8/1)
Focal necrosis (without inflammation)	1 (1/0/0)	0	2 (1/1/0)	3 (2/1/0)
**Respiratory tract (lung)**				**97 lynx**
Mononuclear interstitial pneumonia	38 (17/13/8)	7 (3/4/0)	14 (7/6/1)	59 (27/23/9)
Lungworm-associated granulomatous pneumonia	8 (1/7/0)	3 (2/1/0)	2 (0/1/1)	13 (3/9/1)
Bacterial suppurative bronchopneumonia Others pulmonal changes	4 (4/0/0) 9 (6/1/2)	2 (0/2/0) 2 (2/1/0)	2 (0/2/0) 6 (5/1/0)	8 (4/4/0) 17 (13/3/2)
**Digestive tract** ^1^				**82 lynx**
Gastritis	10 (6/2/2)	10 (1/9/0)	9 (3/6/0)	28 (10/17/2)
Hepatitis	6 (3/3/0)	1 (0/1/0)	3 (1/2/0)	10 (4/6/0)
Others digestive changes	31 (18/8/5)	5 (4/1/0)	8 (6/2/0)	44 (28/11/5)
**Urogenital system** ^1^				**74 lynx**
Interstitial Nephritis	6 (3/2/1)	8 (0/7/1)	12 (2/8/1)	25 (5/17/3)
Membranous glomerulopathy	3 (2/0/1)	5 (0/5/0)	18 (9/7/2)	26 (10/12/3)
Cystitis	2 (1/1/0)	5 (2/3/0)	3 (0/3/0)	10 (3/7/0)
Others urogenital changes	13 (7/2/4)	4 (2/2/0)	5 (4/1/0)	22 (13/5/4)

Juv, juvenile; SA, subadult; Ad, adult.

^1^Some of these degenerative changes and inflammations were present in the same animal.

#### Cardiovascular system.

The most observed incidental findings were related to the heart (125 lynx). Seventy-two lynx showed degenerative changes, 32 had myocarditis and 18 lynx presented both changes. Further three animals showed moderate, acute, focal, myocardial necrosis (without associated inflammation).

Degenerative changes included arteriosclerosis (n = 69), myocardial fibrosis (n = 46) and muscle fiber degenerations with necrosis (n = 14), often in combination; as well as valvular endocardiosis (n = 2). Considering the number of living lynx present in each population, the three lynx populations were equally affected by degenerative cardiac changes (no significant differences). Although these degenerative cardiac changes were found in all age groups (adults: 46/77 (60%); subadults: 21/61 (34%); juveniles: 22/186 (12%)), juveniles and subadults were significantly less affected than adults (odd ratio = 0.09; 95% CI [0.05, 0.17]; p < .001 resp. odd ratio = 0.35; 95% CI [0.17, 0.70]; p = .004).

Inflammatory processes observed included myocarditis (n = 44), epicarditis (n = 3), endocarditis (n = 1) and pericarditis (n = 1). Myocarditis were all mononuclear, predominantly lymphocytic (n = 22) and lymphoplasmacytic (n = 12). Other types of inflammation were histiocytic (n = 4), lymphocytic to histiocytic (n = 3), and plasmacytic to histiocytic (n = 2). Often, these degenerative and inflammatory changes were present in the same animal.

#### Respiratory tract.

The second most frequent incidental findings were related to the respiratory tract (97 lynx), including 80 lynx with pneumonias and 17 individuals with other minor pulmonary changes. Lung changes were mainly mononuclear interstitial pneumonia of unknown cause (n = 59), lungworm-associated granulomatous pneumonia (n = 13) and bacterial suppurative bronchopneumonia (n = 8). The observed interstitial pneumonia consisted commonly of diffuse, interstitial infiltrates of few to moderate numbers of lymphocytes and plasma cells and occasional macrophages. This could be suggestive of viral infection but is an often-observed reaction to a chronic nonspecific insult. Further ancillary testing has commonly not been performed. Lungworm-associated pneumonias were characterized by various inflammatory reactions ranging from interstitial eosinophilic pneumonia to granulomatous or suppurative pneumonia or bronchopneumonia, occasionally with suppurative endarteritis and/or intralesional nematodes (n = 13). Last, the bacterial pneumonias were characterized by neutrophilic inflammation into alveoli and bronchi.

#### Gastrointestinal tract and liver.

Lesions of the digestive tract (from esophagus to anus, including liver and pancreas) were the third most frequently affected organ system (n = 82), including gastritis (n = 28) and hepatitis (n = 10). Three cases of gastritis were related to *Helicobacter*-like organisms, which have been demonstrated by silver staining. Inflammation varied from mild to moderate and variable cell components (neutrophilic to mixed-cellular).

Four gastritis cases appeared to be associated with foreign bodies (hedgehog quills (n = 1), domestic animal ear tag (n = 1) and pieces of wood from a quarantine pen (n = 2)). Nineteen minimal to moderate gastritis cases were presumptively considered secondary to chronic parasitosis based on their cellular composition (eosinophilic (n = 2), eosinophilic to lymphocytic (n = 8) and lymphoplasmacytic (n = 9)). In these animals, infection with *Toxocara cati*, *Capillaria* sp. and/or hookworms was detected coproscopically.

All cases of hepatitis, except of one, were minimal to mild and of distinct inflammatory nature (lymphoplasmacytic (n = 3), necrotizing (n = 3), neutrophilic to histiocytic (n = 2), pyogranulomatous (n = 1), and unspecified (n = 2)). The causes of these inflammatory processes could not be identified.

#### Urogenital tract.

Finally, the fourth most frequent incidental findings concerned the urogenital tract (74 lynx), with mainly membranous glomerulopathy (n = 26, including 11 associated with nephritis), interstitial nephritis (n = 25), and cystitis (n = 10, including 2 associated with nephritis) being diagnosed. Nephritis were predominantly lymphoplasmacytic (n = 16, mild (n = 9) to moderate (n = 7)), not indicative of a specific cause. Other types of inflammatory processes included a lymphocytic (n = 3), lymphocytic to histiocytic (n = 2), histiocytic (n = 1), plasmocytic (n = 1) or mixed (n = 2) component. All cystitis cases, except for one, were mild, and half (n = 5) had an eosinophilic component. This could also suggest a parasitic origin, such as cystitis caused by *Capillaria plica* (Syn: *Pearsonema plica*) [36]. Other urinary bladder inflammations were lymphocytic (n = 2), lymphoplasmacytic (n = 1), neutrophilic (n = 1) and mixed (n = 1).

#### Congenital malformations.

Five non-fatal malformations have been reported, not associated with any other clinical signs or pathologies: a malformation of the sternum and lumbar vertebrae (juvenile female, NES), a malformation of the caudal vertebrae (adult male, ALP), unilateral anophthalmia with hypoplasia of the orbit (subadult male, JUS), ectopic adrenal gland tissue (juvenile female, NES) and ectopic bilateral ureteral orifices (subadult male, JUS).

### Parasites

#### Ectoparasites.

In 151 lynx ectoparasites were reported, and in 38 cases, more than one species of ectoparasites was present in the same animal. Seventy-two lynx were affected by ear mange, 68 by *Otodectes cynotis*, three by *Notoedres cati* and one by both. Fifty-three derived from the JUS population (n = 53/133, 39.8%) and 15 from the ALP (n = 15/182, 8.2%). Lynx with ear mange were more likely to be found in JUS than in the ALP (odd ratio = 9.40; 95% CI [4.97, 19.0]; p < .001) and NES (odd ratio = 6.84; 95% CI [2.26, 29.7]; p = .002).

#### Endoparasites.

Endoparasites were found in 253 out of 315 tested animals (80.3%) macroscopically during post-mortem examination or by coproscopy. Sixty percent of lynx with endoparasites were infected with more than one species (n = 152/253). Parasitized lynx were significantly more frequent than those without endoparasites (*X*^*2*^ (1, N = 315) = 111.01, p < .001). A two-sided binomial test indicated no significant association between body condition (good vs. moderate to poor) and presence of endoparasites (*X*^*2*^ (2, N = 313) = 4.3566, p = .113).

The most frequently detected nematodes by the sedimentation/flotation method were *Toxocara cati* (found in 183 lynx), followed by *Capillaria* sp. (97 lynx) and hookworms (35 lynx). Metastrongyloid lungworms (i.e., *Aelurostrongylus* spp., *Angiostrongylus* spp., and *Troglostrongylus* spp.) could be identified in 16/295 lynx samples analyzed by the Baermann funnel method. Lungworms were significantly more frequently detected in lynx from JUS than from ALP (odd ratio = 4.41; 95% CI [1.50, 16.1]; p = .012)). The presence of *Angiostrongylus vasorum* could be identified by histopathology of lung tissue and molecular methods in one individual examined in 2022. The nematode was observed in a pulmonary blood vessel, associated with a mild-to-moderate interstitial eosinophilic pneumonia.

Only four cases of inflammation of the digestive tract seemed to be related to endoparasites (lymphocytic, eosinophil and eosinophil to lymphoplasmacytic gastritis, respectively; as well as an enteritis with intramuscular granuloma). However, mild inflammation in the gastrointestinal tract was often not recorded if considered as insignificant.

### Miscellaneous incidental findings

A seminoma of the testicles was found at necropsy as incidental finding from a 13-year-old adult male which died of a train accident.

### Additional focus results

#### Radio-collared lynx.

Twenty-eight radio-collared lynx were found dead or culled between 2000 and 2022. Vehicular collisions and traumas of undetermined origins were the two most common COD (n = 5 each), followed by illegal killing (n = 4), and viral infections (n = 4, including three euthanasias due to population sanitary reasons). Other CODs were natural traumas (n = 3), heart failure (n = 2), legal killing because of livestock predation (n = 2), capture for research (drowning following anesthesia) (n = 1) and two remain unknown.

**Fig 7:**
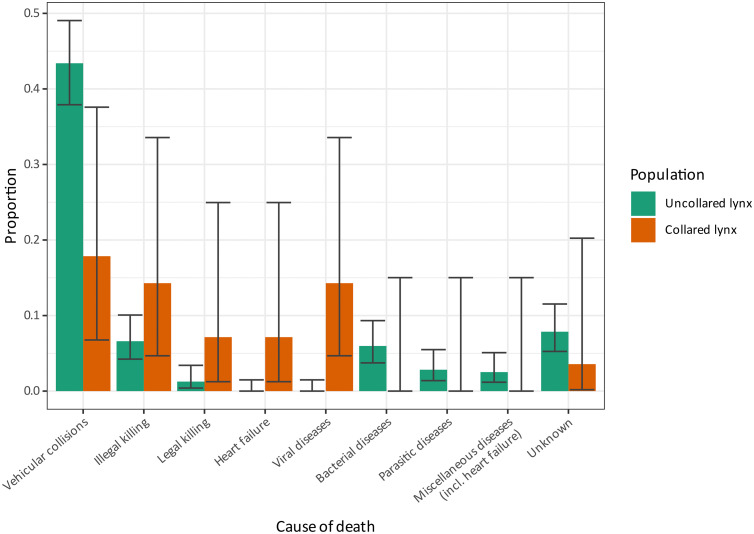
Comparison of causes of death between collared and uncollared lynx in Switzerland, 2000-2022.

Statistical analyses showed that lynx wearing a radio-collar exhibited a different distribution of COD compared to non-collared lynx, with a significantly lower proportion of vehicular collisions (odd ratio = 0.18; 95% CI [0.05, 0.55]; p = .004) and higher proportions of deaths due to miscellaneous traumas (odd ratio = 4.49; 95% CI [1.72, 10.9]; p = 0.001), viral infections (Fisher’s exact test p < .001) and heart failure (Fisher’s exact test p = 0.006) than lynx without (Fig 7).

#### Juvenile lynx.

In total, 194 juveniles between the age of three weeks and 11 months were examined (Table 4). Seventy-six were in good body condition, 28 were in moderate and 81 were in poor body condition. The body condition of nine individuals remained unknown due to advanced decomposition of carcasses.

**Table 4:** Cause of death or reason for culling (inclusive euthanasia) and main pathological findings in juveniles found alive and dead (n = 194), submitted to the Institute for Fish and Wildlife Health, Switzerland, 2000-2022.

COD or reason for culling/euthanasia	Affected animals (n)	Emaciated juveniles	Main pathological findings
Vehicular collisions	70	21	Traumatic lesions (fractures, lacerations, hemorrhages) (n = 70)
Cachexia	54	54	Severe emaciation to cachexia (n = 54)
Miscellaneous traumas	20	11	Traumatic lesions (fractures, lacerations, hemorrhages) (n = 20)
Bacterial infections	14	12	1) Necrotizing and/or suppurative bronchopneumonia (n = 4)^1^ 2) Pseudotuberculosis (n = 3), incl. one lynx with peritonitis, foreign body, and abdominal effusion, necrotizing proctitis with rectal perforation and congenital malformation (lumbar vertebra, pelvis and sternal malformation) (n = 1) 3) Suppurative Pleuropneumonia with pyothorax (n = 2) 4) Septicemia (n = 2) 5) Necrotizing osteomyelitis of the nasal bone (n = 1) 6) Suppurative myocarditis (n = 1) 7) Keratitis and meningoencephalitis (n = 1)
Legally culled orphans in good body conditions^2^	9	0	No pathological findings (n = 9)
Illegal killing	8	3	Traumatic lesions (shot, hit on the head) (n = 8)
Miscellaneous diseases	7	6	Glomerulopathy, with severe soft tissue mineralisations and tubular necrosis (n = 6) Presumptive diaphragmatic malformation (n = 1)
Other anthropogenic traumas	3	1	1) Pulpitis, periodontitis and osteo- myelitis with fistula (n = 1)^3^ 2) Bilateral Fracture of metatarsi, myocarditis, suppurative broncho-pneumonia and fracture with callus (tarsus) (n = 1) ^4^ 3) Multiple bite injuries with laceration of the cervical spine (C1/C2) (n = 1)
Undetermined COD	9	1	

^1^One orphan with a suppurative and necrotizing bronchopneumonia, also showed a catarrhal-suppurative rhinitis, associated with an ulcerative glossitis. A suspicion of feline coryza remained but could not be proven by laboratory analysis.

^2^Nine Juveniles, still in good body condition, were found without their mother near human settlements and culled therefore.

^3^Teeth were damaged due to biting of enclosure walls and metal structures.

^4^This animal was found dead, trapped behind a wood fence in its rehabilitation pen.

The main COD of juveniles was vehicular collision (n = 70), followed by mother’s separation (n = 63), traumas of unknown origin (n = 15), bacterial infection (n = 14) and illegal killing (n = 8). Other CODs included severe soft tissue mineralisation (n = 6), natural traumas (n = 6), captivity-related injuries (n = 2) and accidental trapping in an electric fence (n = 1). The COD of nine individuals could not be determined due to the advanced state of decomposition of the cadavers. The third juvenile who died in captivity, mentioned in the “*Other anthropogenic traumas*” chapter, died at the age of 12 months, and was therefore considered subadult at the time of its death; it is not listed here.

**Fig 8:**
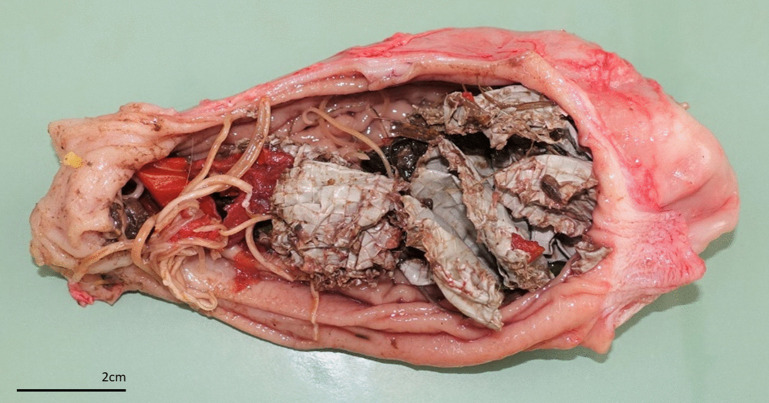
Open stomach of an orphaned lynx with foreign material showing grey plastic waste, tomato pieces and roundworms (*Toxocara cati*).

Macroscopic analysis of stomach contents revealed the presence of food of anthropogenic origin in 28% of presumptive orphans. Fourteen individuals had ingested foreign bodies such as food wrappers, plastics, or fabric (Fig 8), four had ingested human food (such as stew, tomato, or sausage), and three pet food.

## Discussion

### Animal demographics

The number of examined lynx per year has been steadily increasing over the last 23 years, reflecting the populations growth of free-ranging lynx in Switzerland [3].

Juvenile lynx were submitted more frequently than adults. This is probably because separation from their mothers leads them to approach human settlements in search of easily accessible food [15]. There, they are more easily detected and may be legally shot.

### Cause of death

The identification of a COD in more than 90% of the examined animals is high, whereas commonly poor postmortem state hindered the diagnosis in the remaining. The primary COD overall, in radio-collared and uncollared lynx together, was vehicular collision.

The next most frequently observed COD were mother’s separation, miscellaneous traumas, infectious diseases and illegal killing. Other CODs were rare, with neoplasia or degenerative diseases occurring only sporadically.

### Non-infectious diseases

#### Traumas.

As described in other similar European studies [12–14], anthropogenic causes are indeed responsible for most lynx deaths in Switzerland. Vehicular collisions were the leading COD across all ages, sexes, and populations. The proportion of deaths caused by vehicular collisions has increased significantly since the first study from Schmidt-Posthaus et al. [11]. We hypothesized that as the lynx population has increased and expanded over the last 20 years, it is possible that their territories are now closer to human settlements, resulting in higher road mortality. Furthermore, human population and traffic abundance simultaneously expanded [37]. Additionally, in the literature, it is often suggested that the number of animals killed on roads is significantly underestimated. However, in a densely populated country like Switzerland, it is unlikely that a road-killed lynx carcass would remain undiscovered for more than a week, as also any citizens will report carcasses. In fact, this likely results in an overrepresentation of lynx killed by vehicle collisions due to the ease of discovery, compared to other COD.

Subadult lynx and males were significantly more affected by vehicular collisions than other age categories and females. This could be explained by the fact that subadults are on their dispersal and are therefore more likely to cross roads. As males have larger territories than females, they also cover larger distances which might increase their risk for collisions [38].

Lynx from JUS were more frequently affected by vehicular collisions than those from ALP, probably because road density is lower in the Alps [39].

#### Illegal killing and non-lethal attempts.

Twenty-three lynx were illegally killed, and 13 individuals showed evidence of non-lethal, chronic shot injuries, resulting in a total of 36 cases of poaching. Fourteen percent of radio-collared lynx were killed illegally by deliberate shooting or poisoning (14%; 4/28 radio-collared lynx), compared with only 6.3% of dead lynx found by chance (6.3%; 20/318 lynx found by chance). This indicates that poaching of uncollared lynx is very often missed because the carcass is never recovered and submitted for examination. Additionally, even the number of illegally killed radio-collared lynx might still be an underestimate. This is indicated by the recovery of three radio-collars that were cut through. As they were recovered without any carcass the COD of these animals remains speculative and their data were not included in this study. Also, 66 lynx carcasses have not been radiographed, impeding the detection of metal fragments, as fragments are difficult to detect during necropsy only. However, metal fragments were still found in two of these sixty-six lynx during macroscopic examination of the carcass.

Poisons found in necropsied lynx were alpha-chloralose and cyanide. Alpha-chloralose is a central nervous system depressant rodenticide [40]. In the documented case, the animal was found alive with clinical signs similar to those described in cats, such as apathy and tremors [40]. Absorption is rapid and in cats, clinical signs usually occur one to two hours after ingestion but may appear after 15 minutes [40]. The substance is primarily eliminated via the urine after glucuronidation (i.e., by addition of a glucuronide group) in the liver. Therefore, urine should be tested, especially if the analysis of the stomach content is inconclusive as in the case discussed herein. Although alpha-chloralose has rarely been recorded in lynx, this substance is regularly found in wildlife poisoning of free-ranging birds in Switzerland [41] and France [42]. In general, it cannot be ruled out that these rodenticides were targeting rodents. However, their use far from human habitation in the wild remains suspicious for an illegal killing attempt.

Cyanide is one of the fastest acting poisons, causing clinical signs within seconds to minutes of ingestion. Clinical signs observed in lynx were the same as those reported in the literature, including muscle tremors and convulsions, ataxia and severe dyspnea [43]. Poisoned animals die of anoxia, since cyanide inhibits the vital mitochondrial oxidation-reduction reaction in tissues [44].

Compared with shooting, which targets a single individual, poisoning can cause losses in several individuals and various species. The loss of females of breeding age, such as was the case in our study, is particularly damaging, as it means the loss of the whole family group.

In general, our study suggests that both illegal killings of uncollared lynx and failed killing-attempts are underestimated, which is also hypothesized by other authors [45], including the prior study by Schmidt-Posthaus et al. [11]. Illegal killing was the first COD for monitored lynx in Sweden [46], as well as for lynx with or without GPS collars in Croatia [14].

#### Miscellaneous diseases.

Only two radio-collared individuals died of congestive heart failure. Nevertheless, histopathological examination revealed a high proportion of cardiac changes in lynx (124/346, 35.8%), which were accounted as incidental findings. While fibrotic degeneration of the heart and vessels is commonly age-related in domestic animals [47], many juvenile lynx in this study exhibited changes. This raises the question of a possible genetic origin, as described by Ryser et al. [48]. In this study, all three lynx populations (ALP, JUS and NES) seemed to be equally impacted by degenerative cardiac changes. This study also demonstrates that this condition is far more widespread and affects animals of all ages, even if juveniles and subadults are significantly less affected.

Ten lynx were presented with severe soft tissue mineralisation of internal organs mainly in lung, stomach, and kidney, most likely composed of calcium salts. A cause could not be identified in any of the affected individuals. The distribution indicates metastatic mineralisations and thus likely a calcium/phosphor imbalance due to a potential renal insufficiency [49]. A uremic syndrome was also proven to occur in one juvenile brought to a WCC. Other potential causes are a primary or nutritional hyperparathyroidism and hypervitaminosis D. In domestic cats, renal failure is often associated to old age, infections (such as FIV) [50], urolithiasis [51] or ingestion of toxins, such as antifreeze or Vitamin-D-Analogs [52]. However, none of those causes could be linked to the cases described here. Intoxication cannot be completely ruled out: only one animal was tested for the presence of ethylene glycol but neither of the two laboratories involved in this study had the capacity to test for cholecalciferol, used in rodenticide baits. The possibility of a genetic origin is currently being investigated.

Only two cases of tumors were observed in this study: a lymphoma and a seminoma, whereof only the former was related to the death of the animal. Except in a few specific animal species, neoplasia is relatively rare in wildlife [53]. As age is an important risk factor [54], the shorter average lifespan of free-ranging animals compared to captive animals likely limits the occurrence of tumors, as published reports of neoplasia in captive lynx affected animals older than 12 years [55–57].

Although congenital malformations observed by chance in dead lynx over the last 20 years were not considered to be linked to animals’ death, they once again bring up the question of the poor genetic diversity in lynx populations in Switzerland. Ryser et al. (2004) have already raised this question related to malformations observed in live lynx captured as part of translocation projects [48].

#### Infectious diseases.

Infectious diseases represent an increasing threat to wildlife conservation in the world [58], especially for small or isolated populations [59]. Furthermore, transmission of infectious agents from domestic to free-ranging carnivores may become more common in the future as large carnivore habitats become more fragmented and they live in ever closer contact with humans and domestic animals [60]. For example, the fact that lynx in the Jura Mountains show ear mange more often than other lynx populations could potentially be due to a greater proximity to domestic or feral cats in this region of Switzerland (for example through predation). However, in this study, the number of deaths related to infectious diseases was low, although slightly higher among radio-collared lynx. The higher proportion of fatal infectious diseases in collard lynx indicates that these are underrepresented in the passive health surveillance. Some pathogens have already been documented [23,35], but this study is the first to report an infection with *Angiostrongylus vasorum* in lynx.

#### Parasitic diseases.

*Sarcoptes scabiei* was the only ectoparasite of relevance as COD. However, only twelve cases of mange were reported over 20 years, indicating that mange is sporadic and not a major problem for lynx in Switzerland.

Larvae of lungworms were only detected 16 times in lynx feces. In these cases, moderate to severe granulomatous pulmonary lesions were often also found, although these lesions were not fatal. Discrimination of lungworms (i.e., *Aelurostrongylus* spp., *Angiostrongylus* spp., and *Troglostrongylus* spp.) was not routinely performed in all cases as molecular methods are needed to unequivocally identify these nematode larvae to the species level. Such methods were applied in 2022 to confirm the diagnosis of *Angiostrongylus vasorum* in one of the cases. This first report suggests a potential recent introduction of this parasite into the Eurasian lynx population in Switzerland, as it has not been diagnosed in this host before. *Angiostrongylus vasorum* is already widespread, with increasing prevalence in the Swiss fox population [61]. This parasite is also spreading among dogs in endemic regions across Europe [62], possibly facilitated by global warming [63].

Even if endoparasites were present with the same frequency in emaciated and well-nourished lynx, they may contribute to the general weakness of an animal when present in large numbers. *Toxocara cati*, for example, is known to cause stunting in domestic kittens [64]. It would be necessary to analyze the parasite load of these individuals to determine whether diseased lynx have a higher number of parasites than healthy lynx, which is not routinely performed.

#### Bacterial diseases.

Bacterial infections were mainly associated with pneumonia, mostly caused by *Streptococcus canis* (n = 4). This bacterium is opportunistic, resides normally in the upper respiratory tract and can lead to inflammation of several organs, such as respiratory tract and skin in domestic cats [65–66]. It was previously published associated with a fatal myositis in an Iberian lynx [67]. It cannot be ruled out that it was also related to the case of myositis in this study, but the bacterial analyses remained sterile.

Infected wounds were reported five times in this study. In these five cases, skin wounds of varying degrees of severity were suspected to be the entry point for the bacteria, as was already suggested for other wild carnivores such as mustelids [19] or sea otters [68]. Causes of injuries could not always be identified but included one bite wound of a likely co-specific and one illegal gunshot wound.

#### Viral diseases.

As already published by Origgi et al. in 2012 [35], a radio-tagged lynx tested positive for canine distemper for the first time in 2010, followed by a second case one year later. In Switzerland, lynx shares its home range with other carnivore species affected by the virus, such as mustelids [19] or red foxes [69], whereof great losses were observed during the outbreak of 2009–2010 [35].

The described FIV infections were exclusively diagnosed in live lynx that were consequently euthanized. More information to those cases, including the pathological changes have already been published [23]. Thus, our data do not allow any conclusion on the prevalence of retrovirus infections in free-ranging lynx in Switzerland. We suggest to FIWI to integrate retrovirus testing into the postmortem examination protocol.

In one juvenile with a suppurative and necrotizing bronchopneumonia, a catarrhal to suppurative rhinitis and an ulcerative glossitis, a viral cause was highly suspected but could not be proven. Although these lesions are commonly found by feline coryza [70] and the disease has been described in many wild cat species [71], the laboratory analysis (PCR) were negative for herpes- and caliciviruses.

#### Radio-collared lynx.

Radio-collared lynx provide valuable insights into mortality data, as their detection is less affected by carcass detection bias. In non-collared lynx, mortality data are biased toward vehicular trauma due to the higher detectability of carcasses along roads (see above). In contrast, carcasses of radio-collared lynx are more likely to be located rapidly and independently of the cause or location of death. Consequently, their COD are more representative of the population and allow for a more detailed and accurate mortality analysis. In the study of Schmidt-Posthaus et al. [11], infectious diseases were identified as the primary COD for radio-collared lynx, followed by illegal killings. In our study, although infectious diseases were equally found, it seems to be proportionally more frequent in radio-collared lynx than in uncollared ones. However, traumas primarily from vehicular collisions remained the leading COD, followed by illegal killing. The future use of new methods allowing geolocalization of individuals from an early age [72] would greatly improve the currently limited understanding of juvenile mortalities.

#### Juvenile lynx vs orphans.

Overall, 194 juvenile lynx were examined. For one third (n = 63) the COD was compatible with the separation from their mother while two thirds presented with another COD. However, the presence and health status of the mother and potential siblings of an individual are unknown, making the correct classification of these cases challenging. It is possible that lynx became orphans prior to dying from another cause. In juveniles found alive, classification of an orphan status is based on absence of the mother and age (juveniles dependent on their mother until and including March, i.e., before the peak of dispersal [15]). With only the results of a post-mortem examination it is exceedingly difficult to ascertain whether trauma or illness occurred before or after a separation from the mother. For this reason, emaciated juveniles were not classified as orphans based solely on this criterion. It has been documented that a mother may abandon a weak cub, just as a diseased juvenile might be unable to keep up with its mother. However, we strongly suspect that of 37 emaciated juveniles that died from trauma, several of them might have suffered of maternal separation before the traumatic event.

A better knowledge of their diseases may bring some insights to improve management of orphans found alive. It is important to remember that juveniles can be rejected by their mother, or may not be able to follow her due to health problems [73,74]. It is therefore of the utmost importance to ensure that potentially rehabilitated individuals are healthy or, if not, that their impairment is reversible before rehabilitation. Furthermore, the cases of juveniles that did not die despite of, but in consequence of rehabilitation events in unsuitable settings, underlined the importance of quickly available and suitable enclosures as well as expert staff as prerequisites if animals are taken into human care to ensure animal welfare [75].

Although the general increase in population potentially corresponds with the number of orphans, the exact causes of their significant increase remain unknown. More research on the number of individuals per litter, on the weather or on the fate of the mothers is needed to better understand the possible cause of premature separation between a mother and its cub/s.

## Conclusion

Our study provides an update of diseases and COD diagnosed in free-ranging Eurasian lynx in Switzerland from 2000 to 2022 and gives important information on the threats faced by lynx.

Traumas of anthropogenic origin are the most common COD. A significant increase in the number of vehicle collisions has been noted since the study from Schmidt-Posthaus et al. [11]. Considering the ever-growing human population in Switzerland this raises concerns for the future. Efforts to reduce vehicular trauma could include the construction of highway underpasses and green bridges to facilitate wildlife crossing and thus not only reduce mortality but also increase habitat connectivity.

We noted a broad variety of health problems in orphaned lynx and the difficulties that this could cause for their rehabilitation.

Adult lynx were equally diagnosed with various cases of infectious disease, including the first description of *Angiostrongylus vasorum*. However, our evaluation revealed no particular obvious infectious disease that could endanger the lynx population in Switzerland now.

We have reported that infectious diseases, natural trauma, fatal heart failure and illegal killing are proportionally more frequent in radio-collared lynx than in those found by chance. This highlights the importance of this monitoring method to identify the real most frequent COD that seem to endanger the current lynx population in Switzerland. Given that the mortality of uncollared lynx may be underestimated or misinterpreted, further research is crucial, particularly as we suspect a genetic background in two entities. Without a radio-collar, it is difficult to track individual lynx, as they may die in remote areas or be discovered too late for a thorough post-mortem examination. Collars facilitate quicker detection of carcasses, enabling more precise identification of the COD.

Finally, we showed the importance of systemic post-mortem examination and general health surveillance of free-ranging Eurasian lynx in Switzerland. In addition to playing a role in regulating ungulates [76], being part of biodiversity, and being an iconic species in Switzerland, Swiss lynx are valuable in central Europe for various translocation projects. Thus, it is of the utmost importance to closely monitor the health of this species.

## Supporting information

S1 File Regressions. (PDF)
