# Supplementary material for: Causes of morbidity and mortality in free-ranging Eurasian lynx (Lynx lynx) in Switzerland, 2000–2022
Source: PLoS One. 2026 Mar 24;21(3):e0344107. doi: 10.1371/journal.pone.0344107 (PMC13012485; doi:10.1371/journal.pone.0344107)
Supplement: S1 File — (PDF) [file pone.0344107.s001.pdf]

Supplementary material: Regressions

Load packages

```r
library(tidyverse)
```

```
-- Attaching core tidyverse packages ------------------------ tidyverse 2.0.0 --
v dplyr     1.1.4     v readr     2.1.5
v forcats   1.0.0     v stringr   1.5.1
v ggplot2   3.5.1     v tibble    3.2.1
v lubridate 1.9.4     v tidyr     1.3.1
v purrr     1.0.2
-- Conflicts ------------------------------------------ tidyverse_conflicts() --
x dplyr::filter() masks stats::filter()
x dplyr::lag()    masks stats::lag()
i Use the conflicted package (<http://conflicted.r-lib.org/>) to force all conflicts to becor
```

```r
library(tidymodels)
```

```
-- Attaching packages -------------------------------------- tidymodels 1.2.0 --
v broom        1.0.7     v rsample      1.2.1
v dials        1.3.0     v tune         1.2.1
v infer        1.0.7     v workflows    1.1.4
v modeldata    1.4.0     v workflowsets 1.1.0
v parsnip      1.2.1     v yardstick    1.3.1
v recipes      1.1.0
-- Conflicts ----------------------------------------- tidymodels_conflicts() --
x scales::discard() masks purrr::discard()
x dplyr::filter()   masks stats::filter()
x recipes::fixed()  masks stringr::fixed()
x dplyr::lag()      masks stats::lag()
```

```
x yardstick::spec() masks readr::spec()
x recipes::step()   masks stats::step()
* Dig deeper into tidy modeling with R at https://www.tmwr.org
```

```r
library(gtsummary)
```

Vehicular collisions

Factors of interest: Population, Age, Sex and Collar.

```r
vehicule_mod <- parsnip::logistic_reg() |>
  parsnip::set_engine("glm") |>
  parsnip::fit(data = subdf,
               vehicule ~ population + age + sex + collar)

vehicule_summary <- gtsummary::tbl_regression(vehicule_mod,
                                               exponentiate = TRUE,
                                               intercept = TRUE) |>
  gtsummary::add_glance_source_note() |>
  gtsummary::add_q() |>
  gtsummary::add_n(location = "level") |>
  gtsummary::add_nevent(location = "level")
```

```
Extracting {parsnip} model fit with `tbl_regression(x = x$fit, ...)`
```

```r
vehicule_summary
```

Table 1: Summary of the logistic regression with formula: vehicule ~ population + age + sex + collar

Characteristic	N	Event N	OR[1]	95% CI[1]	p-value	q-value[2]
(Intercept)	324	142	0.42	0.21, 0.82	0.012	0.021
population						
ALP	168	59	—	—		
JUS	127	78	2.96	1.78, 4.99	<0.001	<0.001
NES	29	5	0.32	0.10, 0.88	0.040	0.047
age						
Adult	77	28	—	—		
Subadult	61	45	4.04	1.81, 9.34	<0.001	0.003
Juvenile	186	69	0.77	0.40, 1.47	0.4	0.4
sex						
Female	154	58	—	—		
Male	170	84	1.71	1.04, 2.83	0.035	0.047
collar						
No collar	296	137	—	—		
With collar	28	5	0.18	0.05, 0.55	0.004	0.009

[1]OR = Odds Ratio, CI = Confidence Interval

[2]False discovery rate correction for multiple testing

Null deviance = 444; Null df = 323; Log-likelihood = -187; AIC = 388; BIC = 414; Deviance = 374; Residual df = 317; No. Obs. = 324

```
plot(vehicule_summary)
```

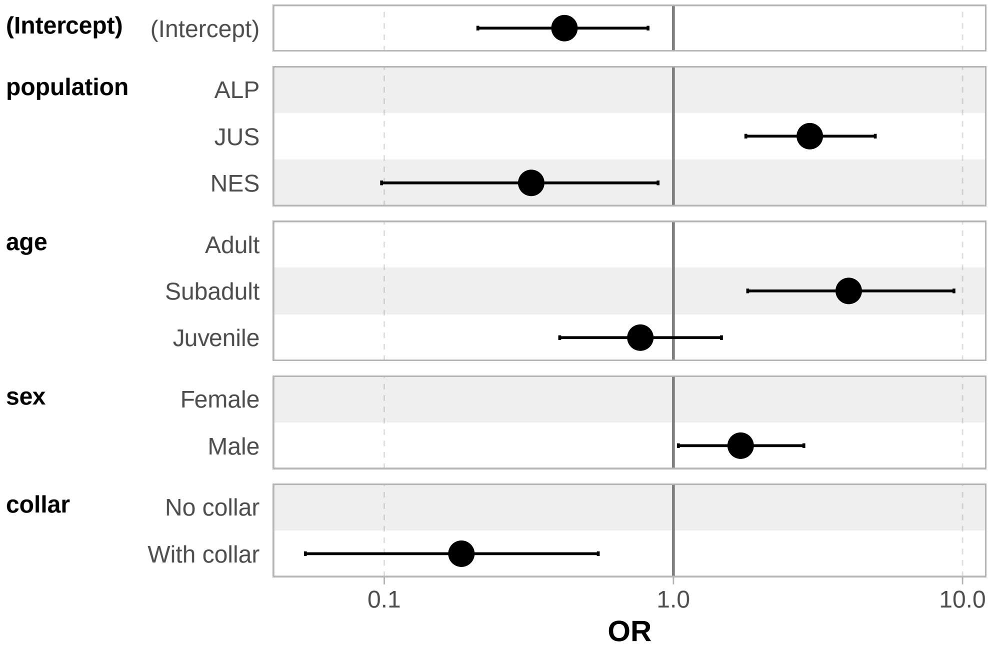

Figure 1: Plot of the output of the logistic regression with formula: vehicule ~ population + age + sex + collar

Illegal killings

Factor of interest: Age

```
illegal_kill_mod <- parsnip::logistic_reg() |>
  parsnip::set_engine("glm") |>
  parsnip::fit(data = subdf,
               illegal_kill ~ age)

illegal_kill_summary <- gtsummary::tbl_regression(illegal_kill_mod,
                                                  exponentiate = TRUE,
                                                  intercept = TRUE) |>
  gtsummary::add_glance_source_note() |>
  gtsummary::add_n(location = "level") |>
  gtsummary::add_nevent(location = "level")
```

```
Extracting {parsnip} model fit with `tbl_regression(x = x$fit, ...)`
```

```
illegal_kill_summary
```

Table 2: Summary of the logistic regression with formula: illegal_kill ~ age

Characteristic	N	Event N	OR[1]	95% CI[1]	p-value
(Intercept)	324	30	0.26	0.15, 0.44	<0.001
age					
Adult	77	16	—	—	
Subadult	61	5	0.34	0.11, 0.93	0.048
Juvenile	186	9	0.19	0.08, 0.45	<0.001

[1]OR = Odds Ratio, CI = Confidence Interval

Null deviance = 200; Null df = 323; Log-likelihood = -92.7; AIC = 191; BIC = 203; Deviance = 185; Residual df = 321; No. Obs. = 324

```
plot(illegal_kill_summary)
```

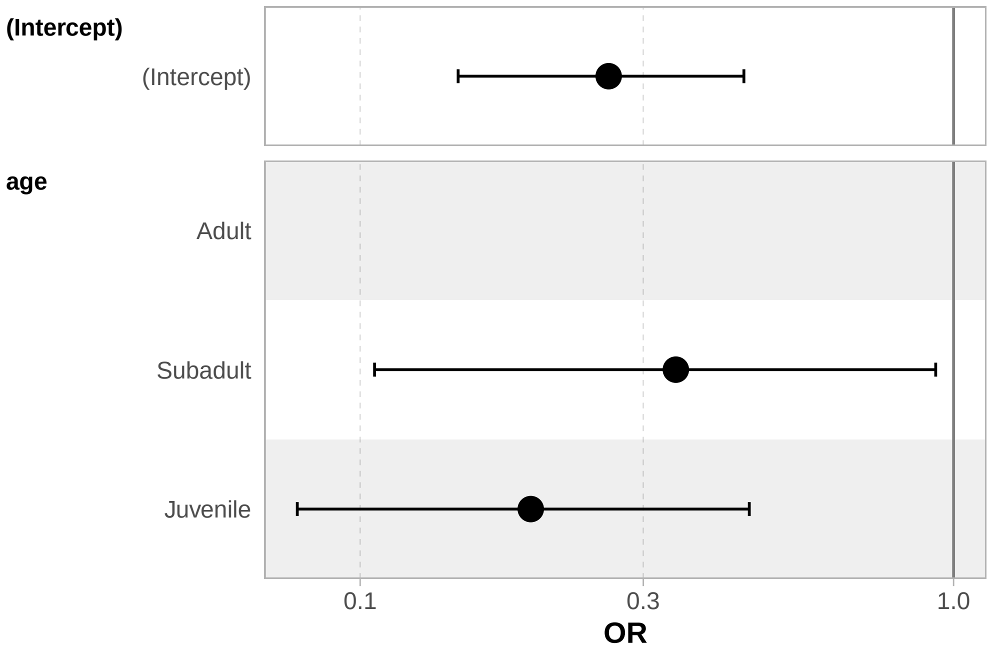

Figure 2: Plot of the output of the logistic regression with formula: illegal_kill ~ age

Cardiovascular system

Factors of interest: Age

```
cardio_mod <- parsnip::logistic_reg() |>
  parsnip::set_engine("glm") |>
  parsnip::fit(data = subdf,
               cardiac ~ age)

cardio_summary <- gtsummary::tbl_regression(cardio_mod,
                                             exponentiate = TRUE,
                                             intercept = TRUE) |>
  gtsummary::add_glance_source_note() |>
  gtsummary::add_n(location = "level") |>
  gtsummary::add_nevent(location = "level")
```

```
Extracting {parsnip} model fit with `tbl_regression(x = x$fit, ...)`
```

```
cardio_summary
```

Table 3: Summary of the logistic regression with formula: cardiac ~ age

Characteristic	N	Event N	OR[1]	95% CI[1]	p-value
(Intercept)	324	89	1.48	0.95, 2.36	0.089
age					
Adult	77	46	—	—	
Subadult	61	21	0.35	0.17, 0.70	0.004
Juvenile	186	22	0.09	0.05, 0.17	<0.001

[1]OR = Odds Ratio, CI = Confidence Interval

Null deviance = 381; Null df = 323; Log-likelihood = -159; AIC = 324; BIC = 335; Deviance = 318; Residual df = 321; No. Obs. = 324

```
plot(cardio_summary)
```

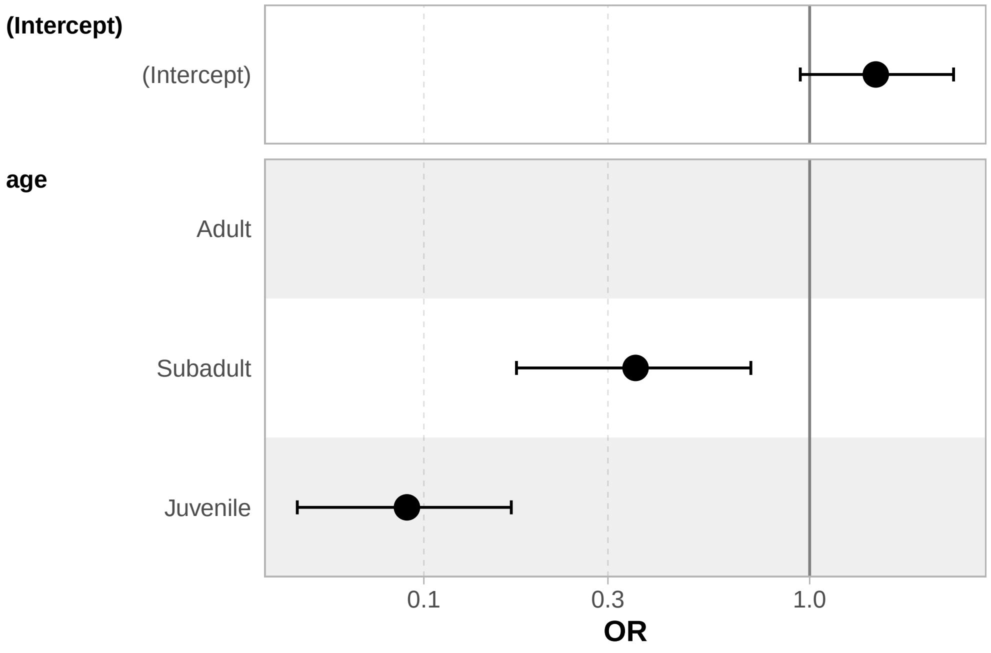

Figure 3: Plot of the output of the logistic regression with formula: cardiac ~ age

Ectoparasites (ear mange)

Factor of interest: Population

```
ear_mange_mod <- parsnip::logistic_reg() |>
  parsnip::set_engine("glm") |>
  parsnip::fit(data = subdf |> filter(! is.na(altitude)),
               ear_mange ~ population)

ear_mange_summary <- gtsummary::tbl_regression(ear_mange_mod,
                                                exponentiate = TRUE,
                                                intercept = TRUE) |>
  gtsummary::add_glance_source_note() |>
  gtsummary::add_n(location = "level") |>
  gtsummary::add_nevent(location = "level")
```

```
Extracting {parsnip} model fit with `tbl_regression(x = x$fit, ...)`
```

```
ear_mange_summary
```

Table 4: Summary of the logistic regression with formula: ear_mange ~ population

**Characteristic**	**N**	**Event N**	**OR**[1]	**95% CI**[1]	**p-value**
(Intercept)	324	72	0.79	0.55, 1.12	0.2
population					
JUS	127	56	—	—	
ALP	168	13	0.11	0.05, 0.20	<0.001
NES	29	3	0.15	0.03, 0.44	0.002

[1]OR = Odds Ratio, CI = Confidence Interval

Null deviance = 343; Null df = 323; Log-likelihood = -143; AIC = 291; BIC = 302; Deviance = 285; Residual df = 321; No. Obs. = 324

```
plot(ear_mange_summary)
```

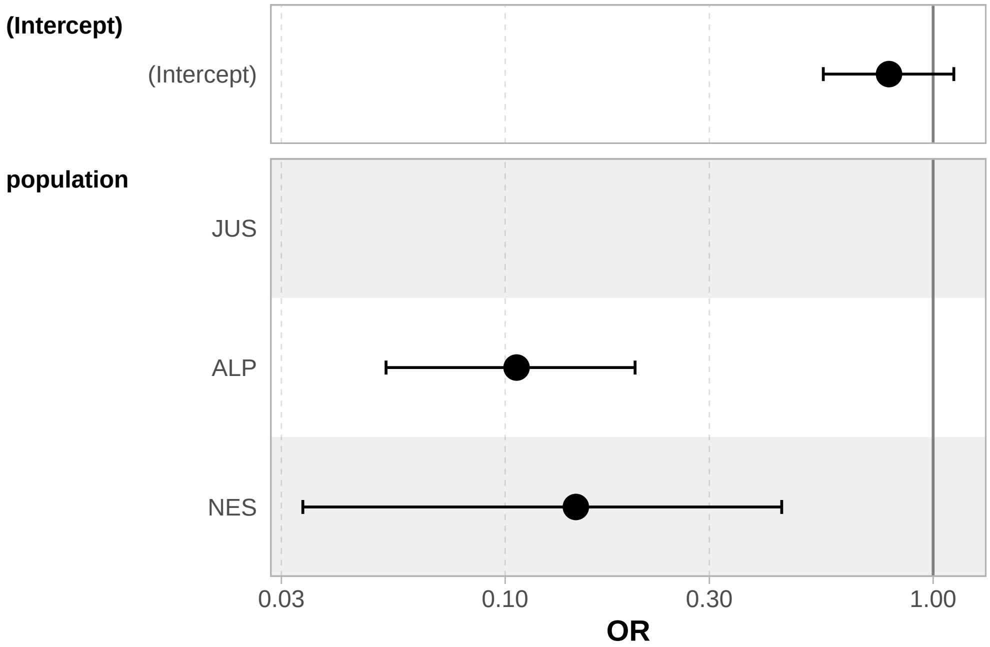

Figure 4: Plot of the output of the logistic regression with formula: ear_mange ~ population

Endoparasites (lungworms)

Factor of interest: Population

```r
lungworms_mod <- parsnip::logistic_reg() |>
  parsnip::set_engine("glm") |>
  parsnip::fit(data = subdf,
               lungworms ~ population)

lungworms_summary <- gtsummary::tbl_regression(lungworms_mod,
                                               exponentiate = TRUE,
                                               intercept = TRUE) |>
  gtsummary::add_glance_source_note() |>
  gtsummary::add_n(location = "level") |>
  gtsummary::add_nevent(location = "level")
```

```
Extracting {parsnip} model fit with `tbl_regression(x = x$fit, ...)`
```

```r
lungworms_summary
```

Table 5: Summary of the logistic regression with formula: lungworms ~ population

**Characteristic**	**N**	**Event N**	**OR**[1]	**95% CI**[1]	**p-value**
(Intercept)	346	17	0.02	0.01, 0.05	<0.001
population					
ALP	182	4	—	—	
JUS	133	12	4.41	1.50, 16.1	0.012
NES	31	1	1.48	0.07, 10.5	0.7

[1]OR = Odds Ratio, CI = Confidence Interval
Null deviance = 136; Null df = 345; Log-likelihood = -64.0; AIC = 134; BIC = 145; Deviance = 128; Residual df = 343; No. Obs. = 346

```r
plot(lungworms_summary)
```

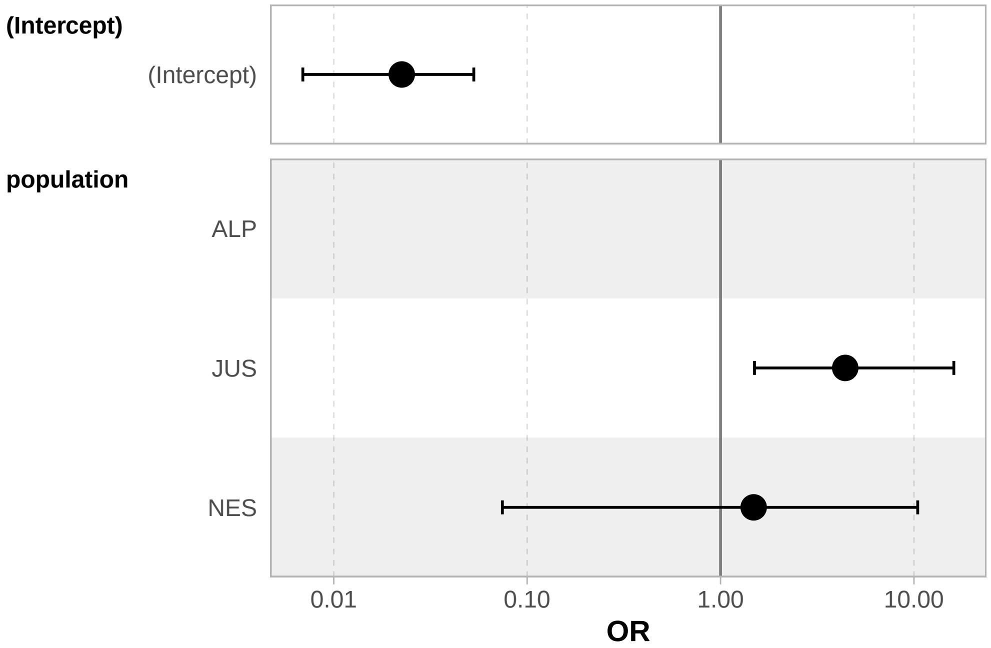

Figure 5: Plot of the output of the logistic regression with formula: lungworms ~ population

Miscellaneous trauma

Factor of interest: Collar

```
trauma_mod <- parsnip::logistic_reg() |>
  parsnip::set_engine("glm") |>
  parsnip::fit(data = subdf,
               trauma ~ collar)

trauma_summary <- gtsummary::tbl_regression(trauma_mod,
                                            exponentiate = TRUE,
                                            intercept = TRUE) |>
  gtsummary::add_glance_source_note() |>
  gtsummary::add_n(location = "level") |>
  gtsummary::add_nevent(location = "level")
```

```
Extracting {parsnip} model fit with `tbl_regression(x = x$fit, ...)`
```

```
trauma_summary
```

Table 6: Summary of the logistic regression with formula: trauma ~ collar

**Characteristic**	**N**	**Event N**	**OR**[1]	**95% CI**[1]	**p-value**
(Intercept)	346	34	0.09	0.06, 0.13	<0.001
collar					
No collar	318	26	—	—	
With collar	28	8	4.49	1.72, 10.9	0.001

[1]OR = Odds Ratio, CI = Confidence Interval
Null deviance = 222; Null df = 345; Log-likelihood = -107; AIC = 218; BIC = 225; Deviance = 214; Residual df = 344; No. Obs. = 346

```
plot(trauma_summary)
```

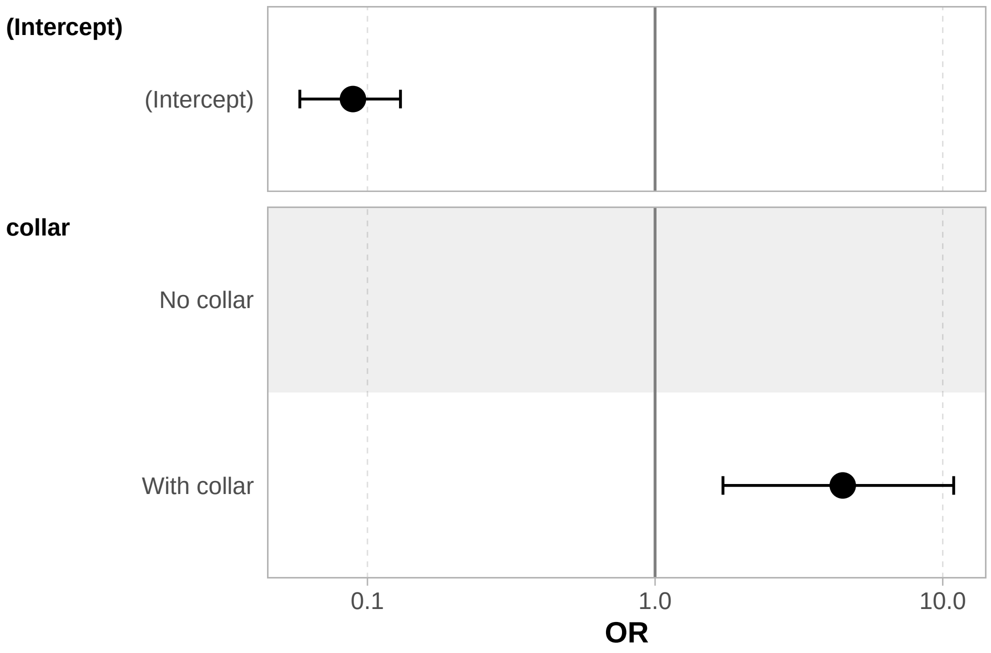

Figure 6: Plot of the output of the logistic regression with formula: ear_mange ~ population
